# Evaluation Method of Relative Humidity Changes in Below-Grade Concrete Structure Space Depending on Different Waterproofing Material and Installation Method

**DOI:** 10.3390/ma13030742

**Published:** 2020-02-06

**Authors:** Ki-won An, Kyu-hwan Oh, Bo Jiang, Xingyang He, Sang-keun Oh

**Affiliations:** 1 Department of Architecture, Graduate School, Seoul National University of Science & Technology, 232 Gongneung-ro, Nowon-gu, Seoul 01811, Korea; ankiwon@seoultech.ac.kr; 2 Department of Railway Construction Engineering, Graduate School of Railway, Seoul National University of Science & Technology, 232 Gongneung-ro, Nowon-gu, Seoul 01811, Korea; kyuhwan.oh@seoultech.ac.kr; 3 School of Civil Engineering, Architecture and Environment, Hubei University of Technology, Wuhan 430064, China; jiangbo15@126.com (B.J.); hxycn@126.com (X.H.); 4 School of Architecture, Seoul National University of Science & Technology, 232 Gongneung-ro, Nowon-gu, Seoul 01811, Korea

**Keywords:** relative humidity, positive-side waterproofing, negative-side waterproofing, below-grade concrete structure space, waterproofing material evaluation

## Abstract

An evaluation method for assessing the difference in the relative humidity (RH) control performance of waterproofing material is proposed. For a demonstration of this evaluation method, two waterproofing materials (urethane coating and cementitious waterproofing material) installed with different methods (positive and negative side of concrete structure respectively) are exposed to temperature conditions representing three seasonal conditions: Summer (40 °C), spring/autumn (20 °C) and winter (4 °C). Condensation level changes on the inner side of the waterproofing material installed specimen is measured, and for derive criteria for comparison, three parameters based on the average RH, intercept RH (derived from a linear regression analysis of RH measurement), and maximum relative humidity are derived for each different waterproofing material installed specimen. Based on quality specification for underground concrete structures, the demonstration evaluation establishes provisional standard criteria of below 70% RH, and all three parameters are evaluated to determine whether the tested waterproofing material/method complies to the performance requirement. Additional analysis through linear regression and cumulative probability density graphs are derived to evaluate the RH consistency and range parameters. The evaluation regime demonstrates a quantitative RH analysis method and apparatus, and a newly designed evaluation criteria is used to compare the RH control performance of positive-side installed urethane waterproofing materials and negative-side installed cementitious waterproofing material.

## 1. Introduction

Many parts of East Asian and South East Asian countries are using below-grade spaces in urban locations for residential apartment building parking lot construction. This heightens social awareness that quality requirements of below-grade construction should be properly analyzed and identified. One of these requirements is humidity control. Generally, relative humidity (RH) control is achieved by installing electrical facilities such as air-conditioners, humidifiers, exhaust fans, and drainage. However, the United States Environmental Protection Agency, in their Moisture Control Guidance for Building Design, Construction and Maintenance outlines humidity control as a requirement for proper waterproofing as well. It is already a well-established fact that if the below-grade space is completely sealed from contact with ground water, it significantly enhances the moisture control of structure indoors [1]. However, there are few existing studies which discuss methods for selecting waterproofing materials that are best suited for this purpose. Existing specifications or test methods only focus on the physical properties of waterproofing membranes for selection criteria, and RH control performance is absent as a required property in almost all cases. Chang et al., in a study on the effect of exterior wall (positive-side installed) waterproofing membranes on humidity-temperature of below-grade concrete structure indicates that there are few studies that examine the correlation between RH conditions (condensation) and waterproofing [2].

Waterproofing with humidity control performance can potentially reduce the energy required to maintain optimal moisture and RH indoors without complete reliance on moisture control facilities, which in turn lowers annual electrical and maintenance costs. In this paper, a newly designed evaluation method based on RH change measurement of a simulated concrete structure interior specimens installed with different types of waterproofing method is proposed. By measuring the condensation changes of the interior of the specimen installed with positive and negative side waterproofing under a predetermined ambient condition results can be derived that will provide a difference between the two waterproofing material and methods. The following sections provide the literature review on the degradation mechanisms associated with leakage and RH in underground spaces and discussion on the requirement for a RH control performance evaluation method for waterproofing materials and methods.

### 1.1. Structural Degradation Problems Due to Increase in RH in Concrete Structures

**Figure 1:**
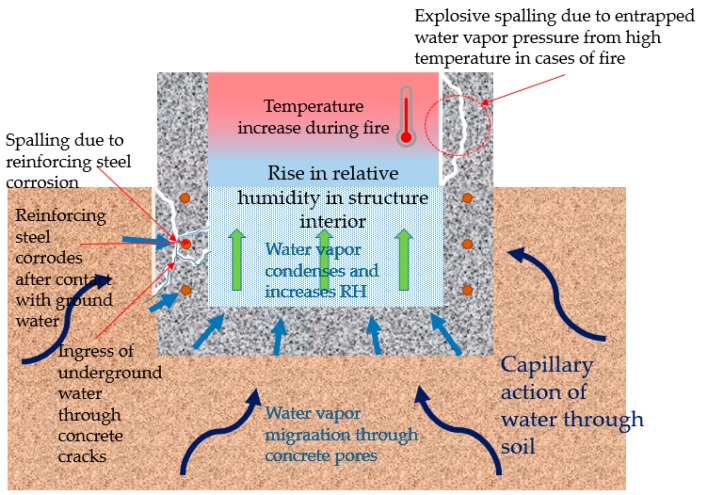
Condensation due to water vapor and water leakage ingress in below-grade concrete structures.

Soils have characteristic capillary action, and water vapor migrates through the minute sized pathing in the soil and come in contact with the concrete structure from all direction [3]. Through the capillary action, water that penetrates the pores of the concrete layer condense into droplets once it reaches the interior due the difference in the ambient conditions. This formation of water in the interior of the structure can be a cause of number of sources for degradation, including delamination, mildew, staining and spalling [4]. This paper discusses two forms of moisture related structural degradation; (1) freeze-thawing and reinforcing steel corrosion, (2) spalling in cases of fire due to increased pressure from trapped moisture. Refer to Figure 1 for an illustration of the water ingress process in below grade concrete structures.

#### 1.1.1. Reinforcing Steel Corrosion and Freeze Thaw

In terms of structural durability, saturated concrete substrates are subject to more extreme degrees of freeze-thaw effect due to seasonal cycling, and can result in corrosion of reinforcing steel. Zhu et al. studies the behavior of steel corrosion under freeze-thawing environment and the study results show that freeze-thaw damage aggravates the steel corrosion in concrete. Their studies also reveal that concrete with waterproofing properties are more resistant to corrosion under freeze-thaw conditioning [5]. These factors indicate that humidity and moisture control in the concrete structure is required. Unless the structure undergoes proper maintenance, the feedback cycle of moisture related degradation in concrete structures creates more entry of moisture and RH in the structure interior [6]. RH and condensation are also a cause for corrosion. According to RA Francis and various sources, the minimum RH humidity below which reinforcing steel corrosion in concrete does not occur is around 80% [7].

#### 1.1.2. Spalling due to Pressurized Moisture in Concrete Due to Increase of Temperature (Fire Hazard)

When concrete is exposed extreme high temperature (most common case being fire hazard), explosive spalling can potentially occur [8,9,10]. When residual moisture components in concrete after hydration reaction is heated, the pressure can eventually cause explosive spalling. Robert Jansson refutes this theory that the presence of moisture is a factor for reducing concrete strength which causes the spalling, rather than the increasing steam pressure [11]. According to his findings, presence of moisture in concrete can be a factor that reduces the strength in areas where spalling can occur in the form of surface flaking during fire exposure [11]. In his paper, experimental study on moisture in fire exposed concrete shows the development of a saturated moisture layer in concrete samples seen to spall during large scale fire exposure tests [11]. From the explicated mechanisms, it is evident that concrete exposed to constant moisture, due to either leakage or high RH become susceptible to spalling during fire hazard. To prevent this risk, the importance of optimal waterproofing and RH control performance can be stressed.

### 1.2. Mold Growth, Discomfort, and Radon Due to Increase in RH

Increase of RH and condensation in underground concrete structure space can create number of social issues. These social issues in the present literature is reviewed in the sections below to illustrate the importance of RH control in concrete structures. Three factors are discussed related discomfort, mold growth, and radon exposure.

#### 1.2.1. Mold Growth Due to Increase of RH

**Figure 2:**
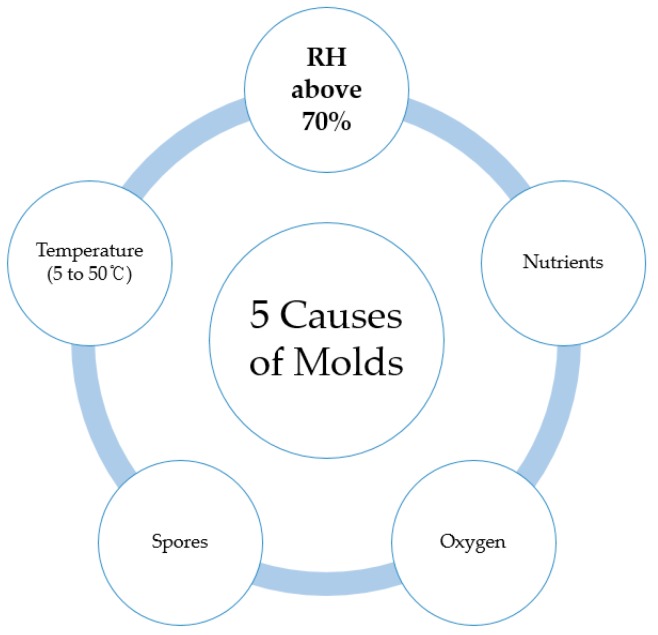
JKR Guidelines on Prevention of Mold Growth in Buildings [6].

Particularly in the case of U.S. and other humid climate regions such as Malaysia and Singapore, it is commonly documented that mold growth in basement can bring significant health risks to residents and in the indoors can produce volatile, bad smelling compounds. The JKR Guidelines on Preventions of Mold Growth in Buildings indicate that when the temperature, nutrient sources, spores, oxygen, and high RH conditions meet a certain level, this leads to mold growth in enclosed spaces [12]. Refer to Figure 2 for details.

Increase of RH in underground concrete structure spaces is mostly attributed to water leakage and poor insulation control. The JKR Guideline outlines the approximate expected RH humidity level for promoting mold growth to be above 70% in conjunction with other biological and ambient factors, and provides a control method ranging from proposed structural designs to architectural considerations [12]. Zuzana et al. provides a report on the varying ranges of moisture level conditions that allow mold growth, but roughly conclude that the critical level lie between the RH of 75% to 95% [13]. Daranee et al. confirm that as hot and humid climate result in a higher risk for mold growth in the indoors of concrete structures [14], RH control should be maintained throughout the year.

In this regard, Johansson et al.’s studies indicate that leakage is identified as a cause for unnecessary increase in RH, but details in the instruction for the selection of material and method is lacking [15]. While there are methods for mold growth prevention, Johansson’s research provides conclusions in that standardized RH control evaluation method is currently lacking in this field.

#### 1.2.2. Discomfort Due to Increase of RH

According to the American Society of Heating, Refrigerating and Air-Conditioning Engineers (ASHRAE), permissible operative temperature range by linear interpolation is between the limits of 0.5 and 1.0 clo. According to the Graphic Comfort Zone Method for Typical Indoor Environments [16]. The basis of the calculation is derived from the predicted mean vote (PMV) that uses heat balance principles to relate six key factors, which include air temperature, radiant temperature, applicable metabolic rate of the residents, clothing insulation, air speed, and humidity. The selected waterproofing method should ensure that the interior humidity and temperature conditions can meet the conditions outlined in the comfort index of ASHRAE, or other comfort zone method systems used as international standards.

Optimal/comfort humidity zone is always relative to the ambient conditions, and it is crucial to be able to examine the humidity control performance in below-grade concrete structures. There are existing sources that provide a reference or guideline on a humidex range and/or criteria, all of which can provide insight on the degree of impact RH have on the quality of concrete structure indoors [16]. However, studies do not commonly mention waterproofing method as a means of controlling RH to maintain the comfort humidity zone. The Environmental Protection Agency guideline *Moisture Control Guidance for Building Design*, *Construction and Maintenance* includes a brief clause on the importance of waterproofing for moisture control of buildings, but detailed specifications on waterproofing methodology is again absent in the guidelines [17].

#### 1.2.3. Health Related Issues Due to RH

Water leakage induced increase of humidity in the interior of buildings can also bring risks of radon ingress. Although radon is normally harmless to the human body when taken in small quantities, enclosed spaces in below-grade structures or basement can result in the radon gas building up. Radon gas is a product of the radioactive decay of radium in natural minerals present in the underground soil, such granite, limestone, and other quarry rocks. The coarse gravel usually laid out before backfilling can also contain uranium that decay into radon particles.

**Figure 3:**
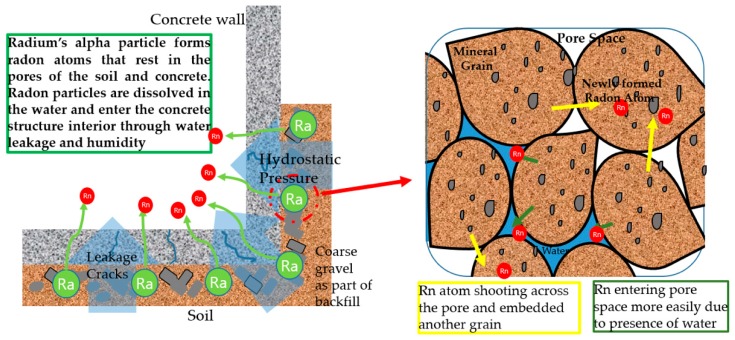
Radon ingress mechanism in below-grade concrete structures.

Radon primarily enters the interior of structures due to air. Scholars such as C.J Groves-Kirkby et al. refute a direct correlation between RH and radon level [18], and active soil depressurization is normally applied to reduce both radon and soil moisture in infiltration [19]. However, high RH caused by the condensation effect due to direct leakage of water can provide indirect entry methods for the radon to enter the concrete interior. According to A. Fernandez-Cortes, the gaseous exchange between surface/underground and atmosphere is linked to the presence of pores and subsurface cavities, which act as storage or temporary sources of radon, and high degrees of moisture in the pores can dissolve the radon gas and enter the atmosphere through diffusion or advective transport [20]. Bradley Turk also provides a detailed study on the effect of RH on the ingress of radon into the interior of concrete structures [21]. Refer to Figure 3 for an illustration on the common way in which radon enters concrete structure interior.

## 2. Protection and Evaluation Methods

While condensation and moisture related problems have long been known to be a cause for degradation of concrete structures and induce the correlated problems as outlined in the above, this factor has not been considered in many of studies related to waterproofing methods and material performance evaluation. National standards such as British Standard (BS EN 8102) [22], American Society for Testing and Materials (ASTM D7832) [23], or China’s national standard (GB 50108) [24] on underground structure waterproofing guidelines all mention the importance of moisture control, but a method for comparatively evaluating different waterproofing materials and methods is not provided. In this regard, the following sections provide a review on the existing waterproofing materials and methods conventionally used in underground structures, and the respective characteristics are outlined to discuss the possibilities for a comparative evaluation on RH control performance. To illustrate the example of this situation, refer to the below Table 1 for details of the ASTM D 7832 for common performance attributes of waterproofing membrane applied to below-grade walls.

In order to achieve a quality waterproofing protection, this study proposes that humidity measurement changes in a below-grade structure simulated environment should be considered as a required performance attribute. In consideration of the above outlined potential problem mechanisms related to different types of waterproofing installation methods, systematic evaluation criteria, and test method for the selection of waterproofing method and material in new underground concrete structures should be proposed.

### 2.1. Waterproofing in Negative and Positive Side on Underground Structures

**Figure 4:**
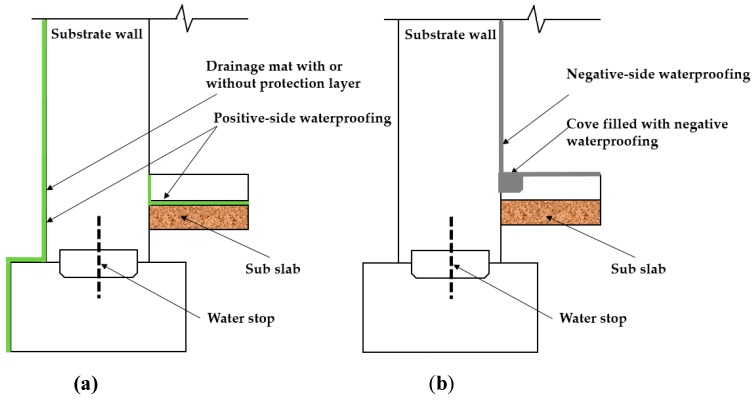
Waterproofing installation methods in below-grade concrete structures [5]; (**a**) positive- side waterproofing design and (**b**) negative-side waterproofing design.

When it comes to the selection of waterproofing materials and methods, there has historically always been concerns with the selection of the material/method for new construction and design to avoid long term problems that concern with structural durability, leakage, and RH level in underground structures. However, as waterproofing construction cost relies mostly one application during construction, rather than material properties, designers, building owners, and constructors are opting to choose materials that are easier to handle during construction to reduce construction time and costs [25]. In this regard, cementitious materials are generally much easier to handle than liquid membrane which is why this material may be used, and performance wise there should not be far too much of a difference when appropriate environmental conditions are met [25]. However, certain waterproofing materials such cementitious materials face various types of problems such as cracking, spalling, and absorption of moisture when used in high temperature/ humidity regions or areas where high water level is expected. Experts may know when to avoid using these materials from experience, but this knowledge is not readily available in a quantifiable data or statistics in an international setting, which is why in many cases, home owners still opt to use materials inappropriate to the construction conditions to reduce construction time/costs. In many cases, negative-side waterproofing is preferred to reduce construction costs and facilitate waterproofing installation [26]. While cost efficiency is certainly a factor for a successful completion of new concrete structure projects, cost reduction may risk reduced quality and durability life-cycle of the structure. Precise identification of environmental degradation factors is not always taken into account, which can lead to selection of inappropriate materials or waterproofing methods. Many of such incidents document cases that resulted in unintended and unexpected rise of maintenance and repair costs that include problems related to reinforcing steel corrosion and water leakage of concrete structures [26]. A concise classification of waterproofing materials in accordance to waterproofing materials applied on different sides of the concrete wall is difficult [27]. Refer to Figure 4 for an illustration on different types of waterproofing installation methods.

#### 2.1.1. Waterproofing Materials Commonly used in Positive Side Waterproofing

**Figure 5:**
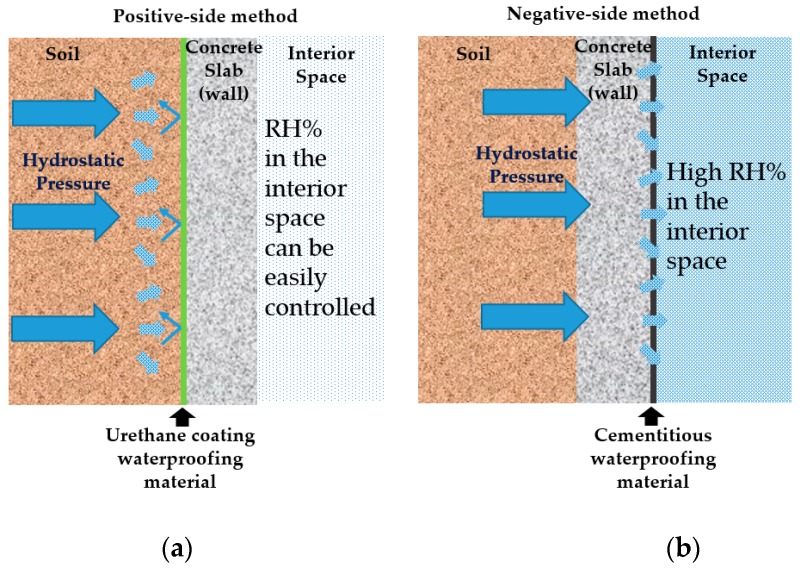
Hydrostatic pressure on the different types of waterproofing methods; (**a**) positive-side waterproofing material (cementitious) and (**b**) negative-side waterproofing (urethane).

For positive side waterproofing system (Refer to Figure 5a)), there are fluid-applied type and sheet membrane type. Most fluid-applied types are advantageous in that they are self-flashing and can be applied in a continuous, seamless layer [28]. Recently developed materials have also resolved previous workability problems related to securing even thickness, the environmental protection policies have prompted the replacement of hazard chemical additives that were originally added into the materials [29]. However, high cost and prolonged construction time are factors to consider, especially in below-grade construction where quality installation is dependent highly on skilled and experienced workmanship [30]. Sheet membrane systems, on the other hand, are generally cheaper and quicker to install compared to fluid-applied type systems, but require more detailed attention for vertical spaces, protruded areas, and small confined areas. Seams and overlap sections are often more vulnerable to degradation and prone to forming leakage paths, and the quality of the waterproofing performance is highly dependent on the condition of the seam or overlap installation sections, thus reinforcing work is more emphasized in these areas [30]. For this demonstration study, a fluid-applied type urethane waterproofing material with the least amount of inconsistent variable for installation was selected for evaluation. Material type selected is compliant to the standard performance criteria of KS F 3211.

#### 2.1.2. Waterproofing Materials Commonly used in Negative Side Waterproofing

For negative side waterproofing system (Refer to Figure 5b)), there are capillary systems and acrylic modified cementitious waterproofing materials [30]. Cementitious waterproofing materials or capillary systems (henceforth called cementitious waterproofing) are applied through coating, and the impregnation into the pores of the concrete substrate matrix allows an integrated installation that provides a water repellent property to the concrete substrate [29]. Cementitious waterproofing materials allow the intrusion of water into the concrete pores as the hydrophilic reaction is necessary for admixtures to close off the pores in the concrete. While this forms a protection against hydrostatic pressure, when saturated these systems are subject to risk of freeze-thaw degradation effects. The acrylic modified cementitious type coating (henceforth called cementitious type) is most commonly used. Installation is comprised of brush or trowel application on concrete or masonry surfaces [28]. Cementitious type materials have strong bonding mechanism even on wet substrate surface [28]. However, cementitious systems lack crack bridging and elastomeric properties, and below-grade concrete structures are often subject to freeze-thaw cycling and structural settlement, and cause movement in joints and cracks [29].

### 2.2. Proposal of Evaluation Criteria Based on Risk Factors Due to High RH in Concrete Structures

The factors discussed in the above stresses the importance of evaluating the condensation level of concrete structures, and the potential to mitigate these risks through selection of optimal waterproofing material and method. As such evaluation method has yet to be devised, this paper proposes such method. The following sections outline demonstrates an evaluation regime of assessing the RH control performance of waterproofing material and method (positive side installed liquid applied membrane and negative side installed cementitious waterproofing material) installed onto an underground structure. The humidity control performance is evaluated in compliance to a RH limit of 70%* in accordance to the KOREA LAND & HOUSING CORPORATION(LH) building specification in Korea [31]. Furthermore, a relative comparison of performance criteria with regards to maintenance capacity of RH through a linear regression analysis of the measured data, and a cumulative probability density analysis to derive the approximate maximum and minimum range of RH for each waterproofing material and method is used to conduct a comprehensive comparison of RH control performance. (* Note: Should this evaluation method be adopted elsewhere, performance criteria for RH limit should accord the relevant national standards.)

## 3. Proposed Evaluation Method for RH Control Performance of Waterproofing Materials

In this study, an evaluation method was designed to observe and analyze the RH changes of the negative side of the concrete wall in accordance to different waterproofing method after applying a fixed temperatures compliant to seasonal characteristics. In this demonstration, the average, intercept derived from linear regression analysis of RH measurement and maximum RH are used as the means to derive an evaluation criterion, and these parameters compared between specimens installed with different waterproofing conditions. First, a mortar substrate specimen untreated with waterproofing is used as an obvious base of verification that waterproofing protection in concrete substrates affect the humidity level in the interior, and a basis for comparison with the specimens installed with waterproofing materials. The waterproofing material installed specimen are prepared in the following respective conditions; (1) positive-side applied liquid membrane and (2) negative-side applied cementitious coating. Based on the results, an assessment will be conducted to state which waterproofing materials and method type can provide the most adequate conditions for humidity control for quality maintenance purposes.

### 3.1. Experimental Equipment

For the temperature and humidity measurement equipment, a sensor that can measure in 0–1 VDC, 0–5 VDC, and 0–10 VDC ranges was used. The sensor was connected to a multichannel data logger wherein the RH level could be measured. The specifications of the sensor and data logger used in the testing are shown in Table 2 and Table 3, respectively, below.

### 3.2. Specimen Assembly for Evaluation

#### 3.2.1. Waterproofing Specimen

Waterproofing specimens were designed to simulate the respective waterproofing installation methods of positive and negative side waterproofing. Specimen substrate was prepared with mortar (W/C ratio: 55%) in accordance to the method outlined in KS L 5207. A Ø100 mm by 80 mm mold is prepared, and fresh mortar is cast into the mold, and cured in the laboratory for 24 hours under the ambient conditions (20 ± 2 °C, 65% RH). The mold is removed from the hardened mortar and is then placed in water for seven days at 20 ± 3°C. Next, the mortar substrate is placed in the temperature chamber again, the ambient condition set at 20 ± 3 °C, 65% RH. Once the mortar substrate is fully cured, the waterproofing materials are installed on to the respective side of the waterproofing installation specification.

**Figure 6:**
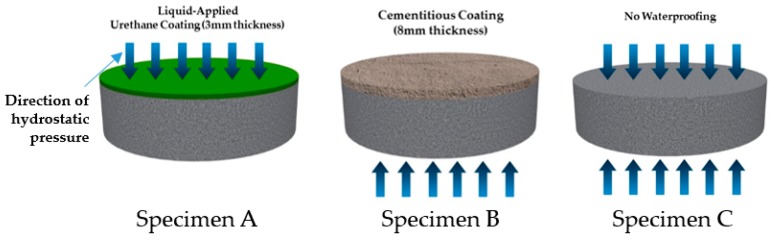
Waterproofing specimens respectively installed with waterproofing materials.

The three types of specimen (Specimens A, B, and C) were made for evaluation. For Specimen A, 3 mm thick layer fluid-applied type urethane waterproofing material (coating) was installed and for Specimen B, 8 mm thick layer cementitious waterproofing material (coating) was applied (among the existing thickness range for waterproofing layer installation specifications, the maximum thickness was selected to ensure the highest quality application condition for the respective material type). For this demonstration study, cementitious type material with the least amount of inconsistent (with factors including mixture ratio, usage cases based on domestic survey, application method taken into consideration) variable for installation was selected for evaluation. Material type selected is compliant to the standard performance criteria of KS F 4919. For Specimen C, waterproofing membrane was not installed. Specimen C was specifically studied and evaluated for reference purposes to display the results of what would happen with the RH level in the negative side of the wall of waterproofing materials are not used. Refer to Figure 6 below for illustration.

#### 3.2.2. Acrylic Apparatus Assembly of Waterproofing Specimens for RH Measurement

**Figure 7:**
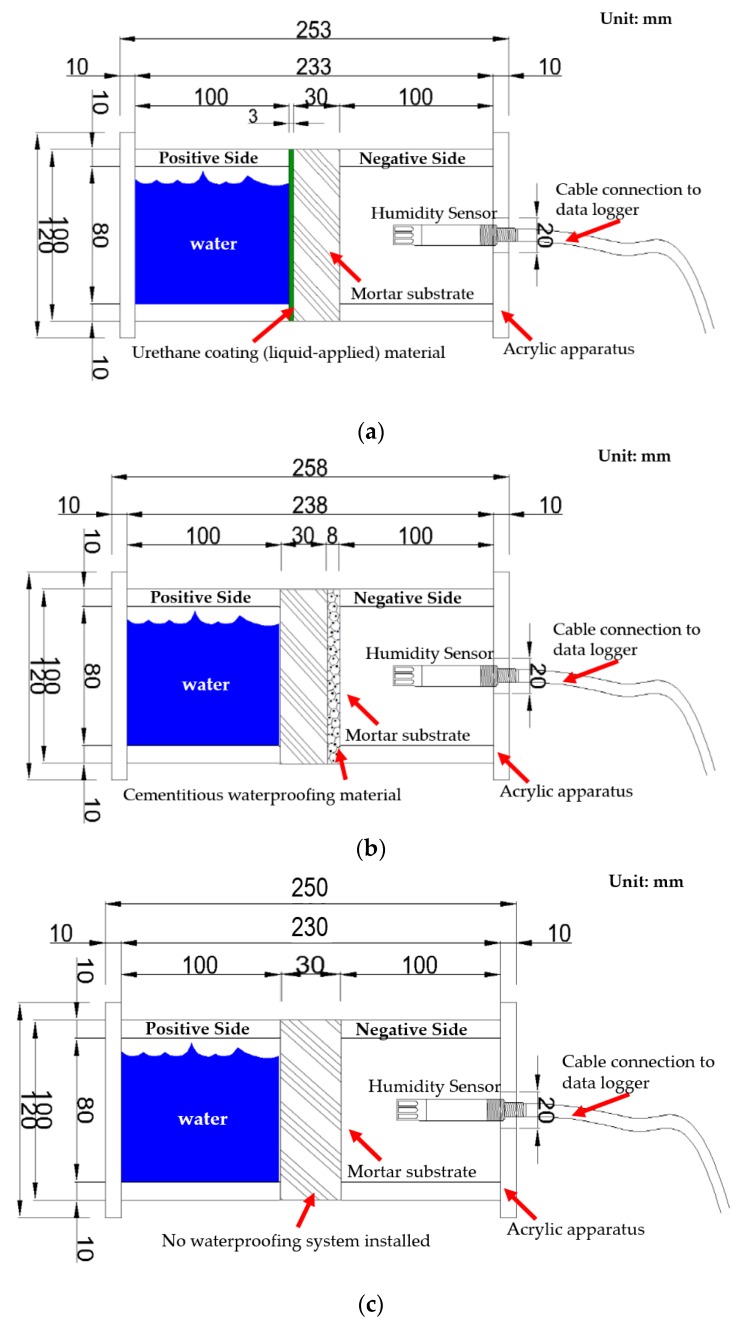
Illustration and dimensions of apparatus of waterproofing specimen; (**a**) Specimen A apparatus: Urethane coating (liquid applied), (**b**) Specimen B apparatus: Cementitious coating, and (**c**) Specimen C apparatus: Untreated.

The three types of specimen are placed between the acrylic apparatus, and the seam joints are covered with silicone sealants. The designated side of the humidity measurement acrylic apparatus representing the negative side of the structure is covered with a metallic cover. On the other side of the acrylic apparatus structure, the metallic cover has a 1 cm hole in the center through which the temperature and humidity sensor is installed. The sensor is then connected to the data logger, and the RH measurement data taken and recorded for analysis. Refer to the below Figure 7 for illustration.

### 3.3. Test Condition Setting for Estimation of Humidity Change Performance of Waterproofing Materials

For this evaluation demonstration, the temperature conditioning for the testing was set in accordance to the average temperatures of winter, spring/autumn, and summer season respectively, and the variable was in compliance to the statistics derived from Korea Meteorological Administration on Monthly Weather Report in Korea 2018 [32].

(1) Winter season temperature for test conditioning (4 °C)

The average temperature during winter season (from December to February [32]) ranges from subzero −15 °C to −10 °C. However, according to the “Standard Specification of Building Construction,” public below-grade structure interior conditions are required to be above 4 °C at all times to avoid the freezing point of water. Therefore, in this study, the temperature conditioning for testing was set at 4°C for winter temperature conditioning.

(2) Spring and fall season temperature for test conditioning (20 °C)

The average temperature during spring and fall seasons ranges between 15–20 °C during spring (March to May) and autumn season (September to November) [32]. Therefore, in this study, the temperature conditioning for testing was set at 20 °C for both the spring and autumn.

(3) Summer season temperature for test conditioning (40 °C)

The average temperature during summer season ranges between 30–40 °C during the summer season (June to August) [32]. To simulate the highest possible temperature condition that can be found in Korea for this study, the temperature conditioning for testing was set at 40 °C. (Note: The conditions outlined above was selected out of lack of standardized regulation for temperature conditions for testing and only serves as a reference value. If this evaluation method is to be conducted elsewhere, Temperature values are subject to change according to different national standards. Examples may include warmer countries with ranges that can reach up to (27 ± 2) °C and colder countries at (16 ± 3) °C, etc. The same applied to humidity conditions.)

### 3.4. Data Collection for the Acrylic Apparatus

**Figure 8:**
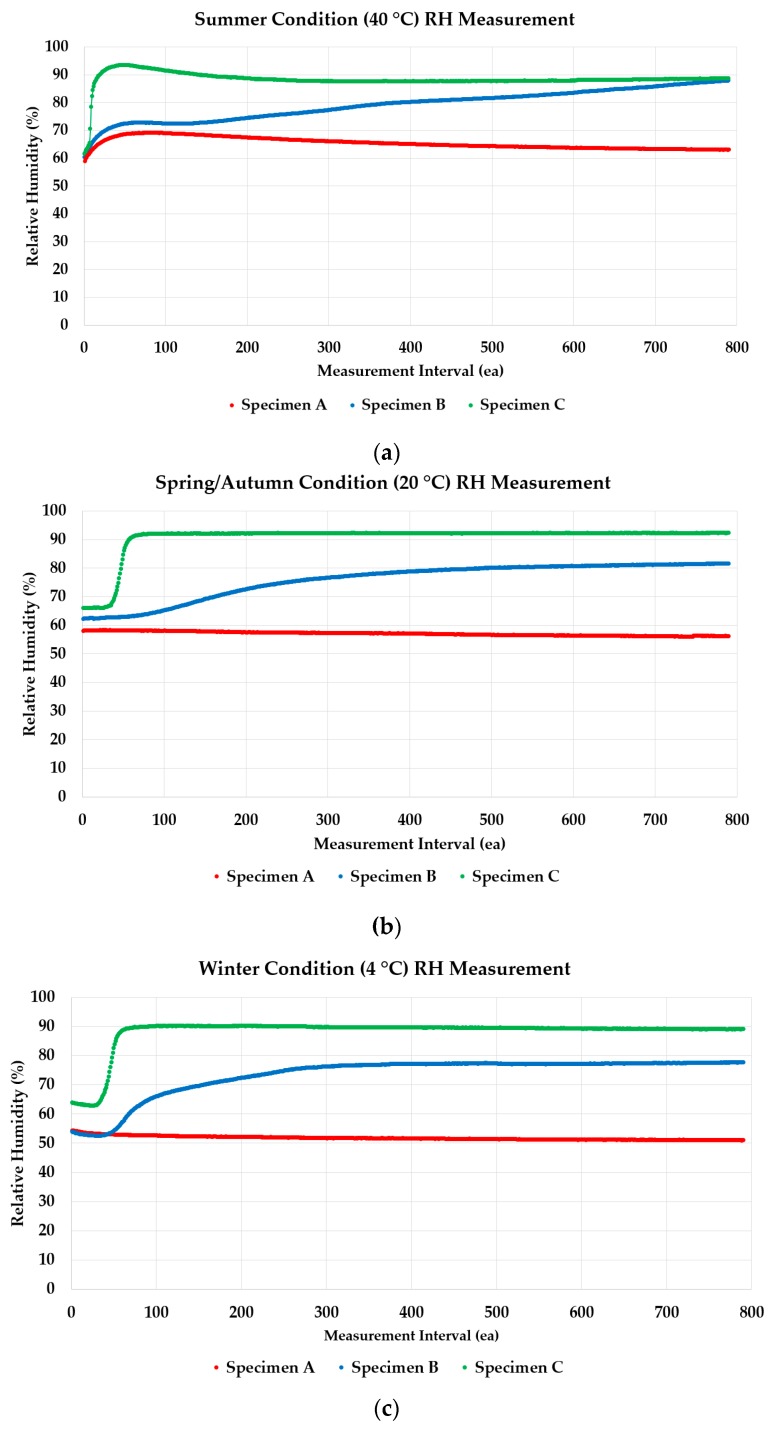
Test method demonstration experimental results; (**a**) winter temperature condition results, (**b**) autumn/spring temperature condition results, and (**c**) summer temperature condition results.

Test apparatus of waterproofing specimens (refer to Figure 8) were placed inside chamber with temperature conditioning set in accordance to the respective seasons (winter, spring/autumn, and summer). The humidity change was recorded in a data logger whose collection interval rate was set to be once every 1200 s*.

#### 3.4.1. Humidity Level Changes of Waterproofing Membrane Installation Methods

The humidity level changes over 798 intervals was measured, and the data was collected using the data logger connected to the humidity sensor. The humidity measurements of the three types of installed specimens were expressed in a line graph in accordance to the seasonal temperature parameters for a) winter, b) spring and autumn, and c) summer in Figure 8, and the results clearly showed the difference in RH conditions of each waterproofing membrane installation methods.

In the winter season (4 °C) condition (refer to Figure 8a)), there was very little change of RH with Specimen A with a declining trend, starting at approximately 53%~55% RH range. In the case of Specimen B, a moderate rise in the RH could be observed at the starting interval during the testing period. This increase was observed approximately between from 0 to 300^th^ interval, and the RH range stabilized, staying at around 7%6–77%. In the case of Specimen C, even sharper increase of RH was detected, from 40^th^ measurement interval to 60^th^, and stabilizing at a high RH range of approximately 90% until the end of the testing period.

In the spring/autumn season (20 °C) condition (refer to Figure 8b)), a very similar pattern was observed for all specimen types, but RH stabilizing point was different with Specimen A and B. Specimen A RH measurement stabilized at approximately 57%–58% range, while with Specimen B the RH stabilization point began more closely at the 400^th^ measurement interval, at approximately 80%. In the case of Specimen C, the RH range stayed at approximately 90% again from the beginning (with a sharp rise at the start) to the end of the testing period.

In the summer season (40 °C) condition (refer to Figure 8c)), the starting RH measurement trends were observed to be more curved, with an increase of RH to approximately 70% and decreasing stabilization of 63%–64% with Specimen A. In the case of Specimen B however, a distinct trend of increase in RH was observed, eventually reaching the same level of RH of approximately 90% as with Specimen C from the 750^th^ measurement interval and onwards.

Overall, the RH of the Specimen C was the highest, reaching on average 77%–90% during all seasons. Specimen A was able to maintain a stable RH% below 70% in all three seasonal temperatures. In the case of Specimen B however, it was shown that in the winter season, the RH measurement showed a steady increase from a below 70% to as much as above 80%.

From this analysis results, Specimen A would be the only waterproofing material and installation type that can meet the requirement of the LH Building Construction specification standard criteria.

While this was an expected result with Specimen C as it was untreated with a waterproofing material, Specimen B was able to demonstrate some level of RH humidity control performance in comparison due to the waterproofing layer, and the increase in the RH was not as apparent. However, in high temperature condition, the RH eventually reached the same level as it would with the case of Specimen C. This phenomenon expectedly indicates that cementitious waterproofing material and untreated substrate layer allows almost immediate moisture penetration into the interior space without any reinforcing insulation layer, as there is an obvious indication that the saturation of water in the substrate affected the interior RH level. In comparison, Specimen A maintained the lowest RH throughout all of the seasonal temperature conditioning.

**Figure 9:**
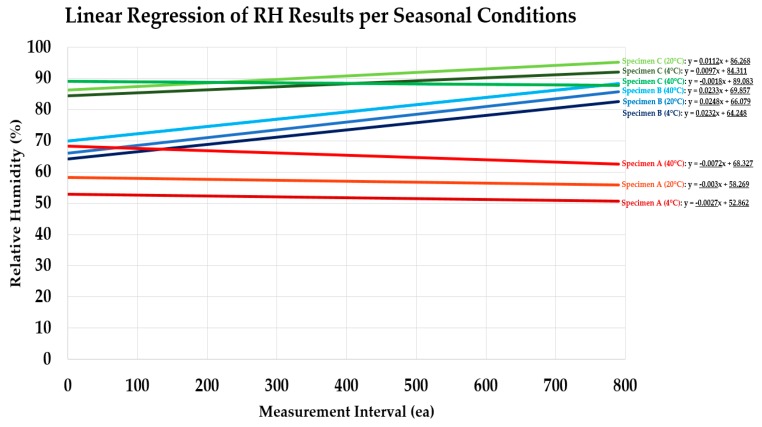
Slope calculation through regression line of relative humidity (RH) measurement results.

To analyze the RH control performance with regards to changing RH throughout the testing period, a linear regression graph was derived to analyze the RH maintenance tendency for each specimen type under the respective seasonal temperature conditions. The maintenance tendency is determined by the slope and the intercept values obtained from the regression lines and this performance trait is compared between the specimen types. Refer to Figure 9 and Table 4 below for details.

According to the slope derivation results, every specimen had a relatively consistent slopes, but some distinct tendencies could still be observed for each specimen types. Specimen A had a declining slope for all three seasonal conditions. This result could indicate that the RH can be expected to be easily controlled owing to the observation that the RH level stabilizes and decreases overtime despite the continued exposure to water at the positive side of the substrate. In contrast, Specimen B had inclining slopes for all seasonal conditions, indicating that even though initial RH may be similar to that of Specimen A, increase of RH in the interior space can be expected. In case of Specimen C, varying results from declining to inclining slopes were observed, but from comparing the intercept values, the RH level was far above the permissible level of RH (in accordance to the LH Building Construction specification). The results verify that positive-side installed urethane coating waterproofing membranes (Specimen A) had the most stable RH maintenance performance, remaining at below the required RH criteria. Whereas with the negative-side installed cementitious waterproofing material, it is expected that eventually the RH will consistently increase overtime due to the exposure of continued hydrostatic pressure.

#### 3.4.2. Parameter Analysis (Average, Slope Intercept, and Maximum) Measured RH and Comparison

Lastly, the average, and maximum RH was calculated from the data intervals derived Figure 8, and the intercept was derived directly from the linear regression line equations from Figure 9 between the results of the three separate seasons, and the recorded results are outlined in Table 5 below.

**Figure 10:**
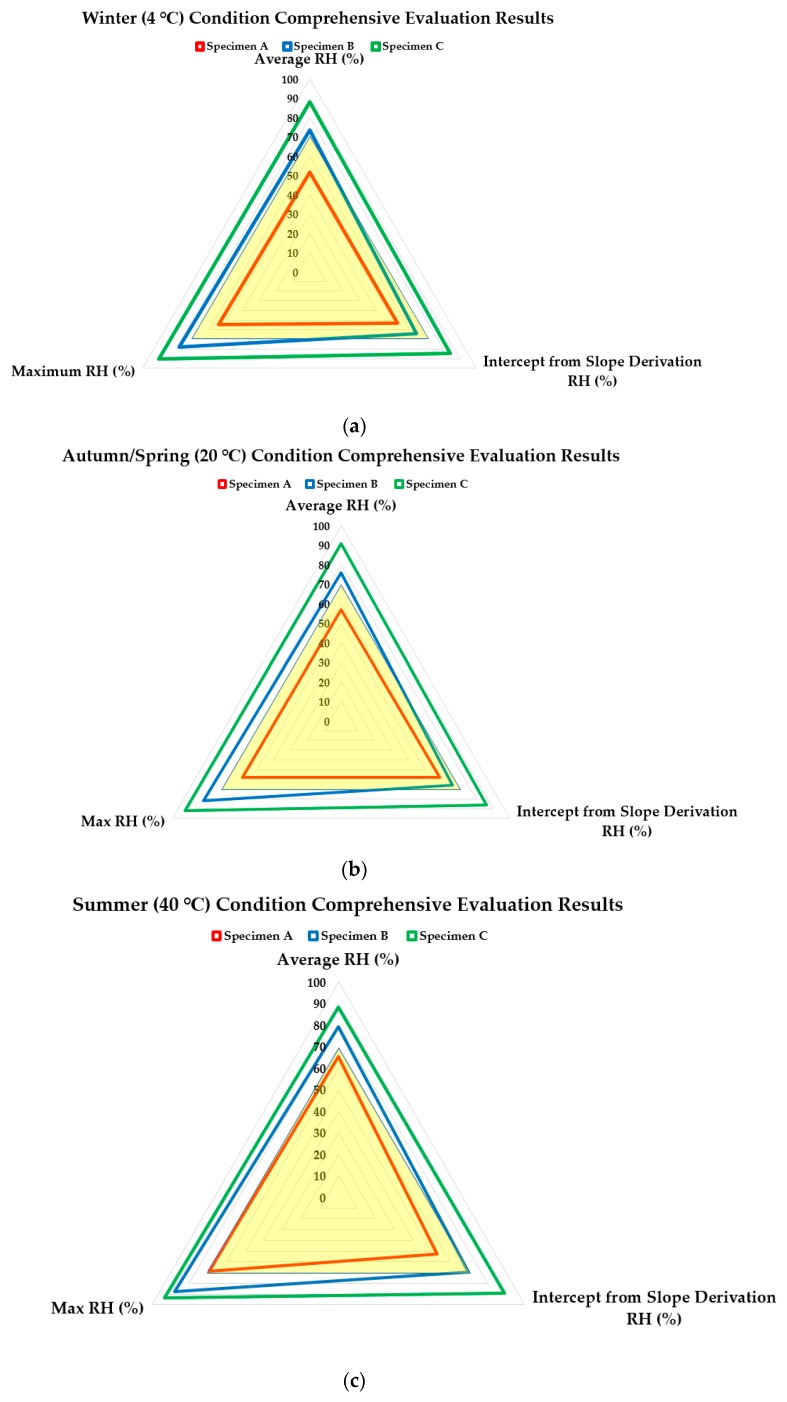

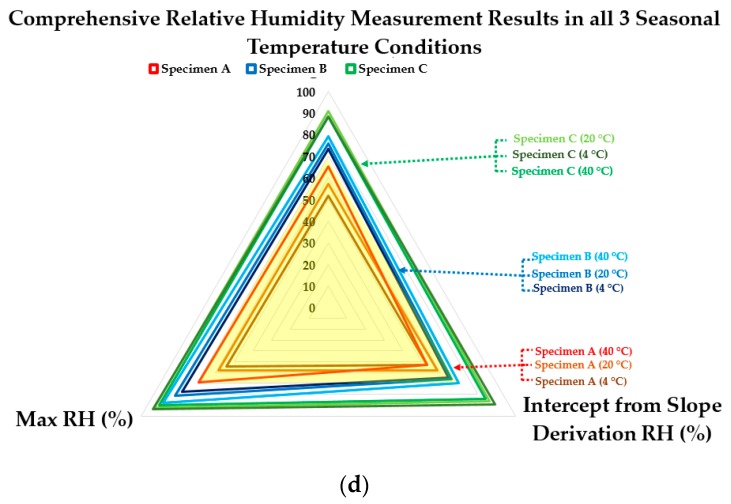
Average, maximum, and intercept (from slope derivation) RH comparison with respect to standard criteria requirement of three specimen types in accordance to different seasonal temperature conditions; (**a**) winter condition results; (**b**) spring/autumn condition results; (**c**) summer condition results; and (**d**) comprehensive comparison graph of the three types of specimens.

Based on the recorded RH change measurement related factors, a comprehensive evaluation was conducted to compare the ease of humidity control in the negative side of the below grade structure based on the waterproofing method used. For this, a radar chart based on the three different factors (average, intercept and maximum) was derived in accordance to different seasonal temperature conditions in Figure 10. The maximum range of the radar chart was set to 100% RH and the recorded data was plotted on the graphs. For each factor, the higher values indicate that the RH change conditions are not moderate, and could indicate difficulty in humidity control using conventional methods using electrical facilities and equipment. The yellow region in all of the radar charts indicate the permissible RH range in accordance to the LH Building Construction specification (below RH level of 70%).

In the winter season (4 °C) condition (refer to Figure 10a)), it was observed that for Specimen A, the RH level with regards to average value (51.78%), slope intercept (52.86%), and maximum RH (54.42%) was all able to be within the 70% RH range limit, complying to the LH Building Construction specification standard criteria. With Specimen B, the average (73.42%) and maximum (77.79%) measured RH were above the 70% RH standard criteria, but the intercept (64.25%) was within the standard criteria range, indicating that throughout the testing period, RH control performance in the earlier period with minimal exposure to water can be expected. In the case of Specimen C, all three parameters were outside of the 70% RH standard criteria (88.14%, 84.31%, and 90.25%, respectively).

In the spring/autumn season (20 °C) condition (refer to Figure 10b)), Specimen A again met the required performance criteria for all three parameters (57.10%, 58.27%, and 58.31%, respectively), and Specimen B was able to meet the standard criteria for the slope intercept (66.08%) again, but the deviation from the standard criteria was marginally higher than the winter condition results, especially in the case with maximum measured RH (81.64%). In the case of Specimen C, all three parameters were again outside of the 70% RH standard criteria (90.69%, 86.27%, and 92.41%, respectively).

In the summer season (40 °C) condition (refer to Figure 10c)), Specimen A met the required performance criteria for all three parameters, but the average and maximum RH were higher (65.47% and 69.16%, respectively). In the case of Specimen B, the RH measurement result for the intercept was barely able to meet the standard criteria (69.86%) with the deviation from the standard criteria was much higher than the previous seasonal temperature conditions (79.06% average RH, and 87.85% maximum RH). In the case of Specimen C, all three parameters were again outside of the 70% RH standard criteria, but the measurement did not increase as much in contrast to the results found with Specimen A and B (88.38%, 89.08%, and 93.43%, respectively).

A comprehensive view (refer to Figure 10d)), of the RH measurement results in all three seasonal temperature conditions with respect to the three parameters show the overall expected RH control performance for the different specimen types. With Specimen A, while a satisfactory performance can be expected throughout the annual cycle, maximum RH value during summer temperature conditions indicate that supplementary RH control is necessary to ensure that the RH level in the interior space can maintain below the standard criteria limit of 70% RH. With Specimen B, it can be inferred from the results that the waterproofing method and material alone is not sufficient to provide a satisfactory RH control performance throughout the year. Manufacturers and designers should take special care to provide the necessary RH control factors in the structures using this type of waterproofing material and method.

Based on the parameter evaluation, comparing the results of Specimen C (untreated) with Specimen A and B affirm that waterproofing layer affects the RH control substantially and comparison of Specimen A (positive-side urethane coating) and Specimen B (negative-side cementitious waterproofing) affirm that different RH control performance can be expected from the respective types of waterproofing material and method.

#### 3.4.3. Probability Density Analysis of Measured RH

For further analysis, a normative distribution analysis of the complete RH measurement intervals (798 intervals) was conducted. To derive the probability density of the RH throughout the entire year (comprehensive data obtained from Figure 9) for the respective types of specimens, a probability density analysis of the RH measurement was conducted using the following function;

$$f\left(x|\mu ,\sigma^{2}\right)=\frac{1}{\sqrt{2\pi \sigma^{2}}}e^{-\frac{{\left(x-\mu \right)}^{2}}{2\sigma^{2}}}$$

where;

*μ* = the mean of distribution of the RH data set

*σ* = standard deviation of the RH data set

*σ*^2^ = variance

Based on the calculation, the following normative distribution graphs were derived for each graph, the results of the three different specimens are displayed (total sample size of 2380 data interval). The deviation range was set in the intervals of 1% range difference of RH, and the frequency for each RH deviation range was displayed in percentage value.

**Figure 11:**
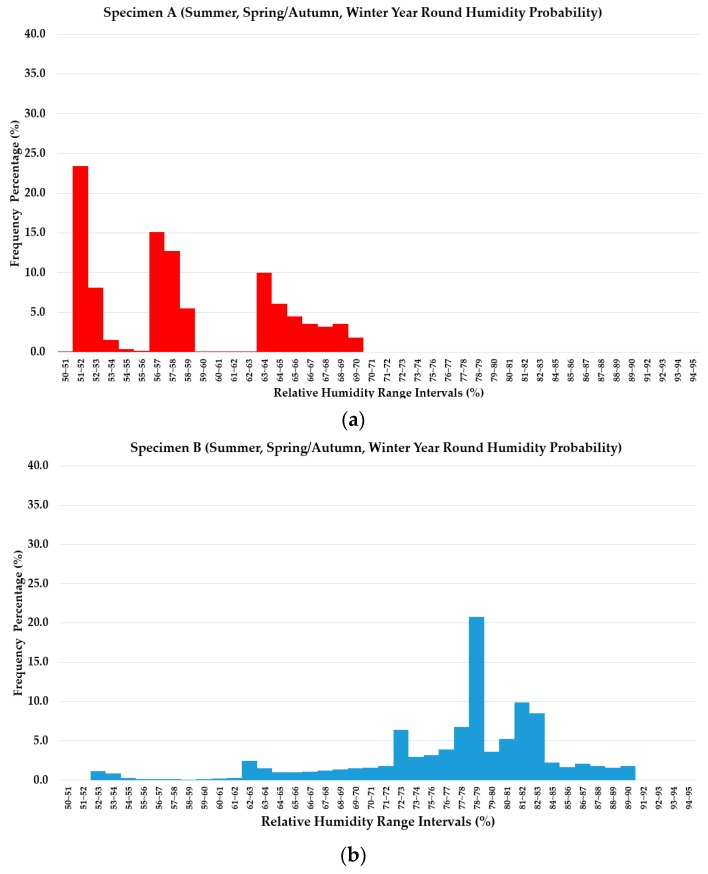

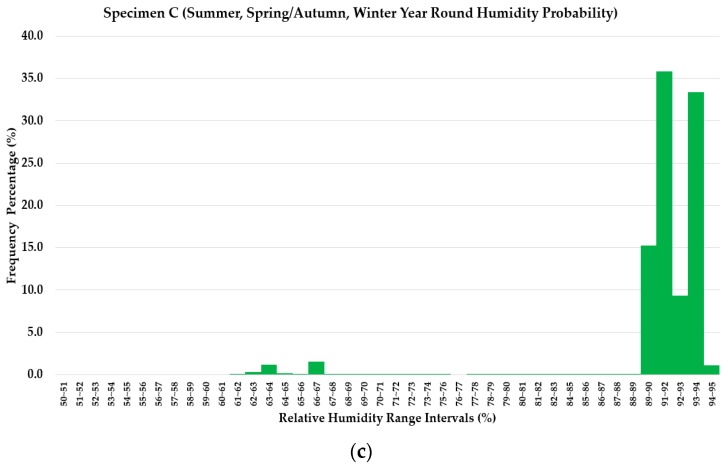
Probability density of RH measurement of three types of specimen throughout the three seasonal changes (year round), (**a**) Specimen A results (**b**) Specimen B results, and (**c**) Specimen C results.

The probability density analysis displays the overall expected range of the measured RH results of the three different specimen types throughout the three seasonal temperature conditions. The above comparison of the three different parameters (average, intercept, and maximum RH) and slopes is more clearly contrasted through observing the height of the peak within the total range. For the results of the probability density analysis, it is observed that for Specimen A, distinct peaks indicate performance visibly varies between the seasons, but overall is able to maintain the RH level below 70% all year round. Expected varying range of RH is from approximately 50%–70% RH, indicating that the Specimen A is able to provide RH control performance satisfactory to the LH Building Construction standard criteria. In the case of Specimen B, a normally distributed graph can be derived based on the RH measurement results of all three seasons, but the range of expected RH varies from low frequency of 52%~53% up to as high as 89%~90% all year round, mostly maintaining the RH of between 78%–79%. In the case of Specimen C (untreated), very low frequency at low RH ranges of 61%–67% could be observed, but the RH range mostly accumulates around the range of 89%–95%. Refer to Figure 11 for details;

With respect the entire evaluation regime proposed in this study, the following Table 6 provides the summary of the performance evaluation results from the analysis process discussed in the previous sections.

The results of this evaluation method demonstration cements the reasoning that waterproofing materials have a significant influence on humidity, as they are the frontier for providing ingress or permeation of water in the concrete substrate. Based on the above observations in the separate seasonal temperature conditioning, it can be seen that Specimen A (urethane coating material) has the highest RH humidity control performance throughout the year, whereas Specimens B and C are not able to meet the standard criteria in comparison. This simplified representation of the respective waterproofing material type and installation methods allows a clearer comparison in terms of the RH control performance when installed in a concrete structure.

## 4. Conclusions

This study proposed a new evaluation method as well as confirming:

(1) that waterproofing material (type and existence of) impacts humidity in the negative side of the concrete structure across different seasonal conditions;
(2) an evaluation of the average, intercept and maximum RH, slope, and the RH variance range can be used to compare the different waterproofing methods and materials’ humidity control performance
(3) the study results provide an insight on how easy future humidity control would be given a waterproofing material.

While this evaluation method requires revisions to better simulate the below-grade structure environment, it serves as a basis for selecting optimal waterproofing materials. Future research will include criteria for optimal humidity conditions for indoor comfort, prevention of radon ingress and mold growth, and a cost efficiency analysis based on electricity usage. Future evaluation using this method will include assessment and comparison results of different types of materials commonly used in underground concrete structures including asphalt or synthetic polymer sheet.

## Figures and Tables

**Table 1:** American Society for Testing and Materials (ASTM D7832) Waterproofing Material Physical Properties [23].

Property	Standard	Criteria
Waterproofing Material Physical Properties for Bituminous and non-bituminous organic		
Resistance to Hydrostatic Pressure	Test Method D5385	No leaks at 103 kPa [15 psi] [34.65 ft head] or at the maximum hydrostatic pressure determined by the subsurface soil investigation per IBC para. 1802.2.3
Resistance to Deterioration from Organisms and Substances in Contacting Soil	Test Method D7281	Pass
	Test Method E154	<0.3 perms water vapor permeability
	Test Specification E1745	Section 7 (using a 3 in. thick precast concrete paver in lieu of cast-in-place concrete).
Adhesion to Substrate (Except for Grade 1, Class 2)	Test Method D7234	>1518 kPa [220 psi] using a 50 mm [2 in.] dolly
	Test Method D903	3 pli [535.8 gm/cm]
Waterproofing Material Physical Properties for bituminous		
Low Temperature Unrolling Type 1A	Test Method D5636	No Cracking at 0°C [32 °F]
Crack Bridging	Test Method D5849	500 cycles select appropriate temperature for the weather conditions for which the membrane is applied.
Flexibility	Test Method D5683	No Cracking
Water Absorption	Test Method D95	<2 % by weight. Run 45 cycles of immersion in water at 23 °C and 50 °C [73 °F and 122 °F] for 24 h for start of test and after 45th cycle.
Waterproofing Material Physical Properties for Non-bituminous Organic		
Water Absorption	Test Method D570	<3 % by weight when tested per Section 7A
	Test Method D471	<3 % by weight when tested per Section 12 at 23 °C [73 °F] for 2998 h
Linear Dimension Change (PVC only)	Specification D4551	<5 % at 70 °C [158°F] 1 h per Test Method D1204
Extensibility After Heat Aging	Test Method C1522	6.4 mm [1⁄4 in.]
Crack Bridging Ability	Test Method C1305	No Cracking
Low Temperature Flexibility and Crack Bridging for Liquid-Applied Membranes	Test Method C1305	Pass
Resistance of Plastics to Bacteria	Specification D4551	No effect 12 of 12 samples pass
Resistance to Chemical Reagents	Practice D896	No delamination, blistering, emulsification, or (undiluted deterioration 15 N/5P/5Potash)
Resistance to Petroleum	Test Methods E154 Section 14	<0.3 perms

**Table 2:** Temperature and humidity sensor specification.

Item	Criteria
RH Range	0~100% RH
Temperature Range	0~100 °C, −20~80 °C
Precision at 23 °C	<±2% RH (10~99% RH)
Annual RH Stability	<±1% RH
RH reaction time	<10 s (up to 90%)
Signal Measurement	0-1 VDC, 0-5 VDC, 0-10 VDC
Size	Length: 80 mm, Ȼ12 mm

**Table 3:** Data logger specification.

Item	Support	Photo
DCV Precision	0.004% / Year	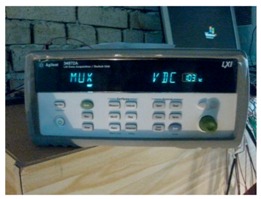
Max. Scanning Speed	250 channels / sec	

**Table 4:** Slopes and intercepts derived through regression line of RH measurement results.

Specimen Types	Seasonal Conditions					
	Winter (4 °C)		Spring/Autumn (20 °C)		Summer (40 °C)	
	Slope	Intercept (%)	Slope	Intercept (%)	Slope	Intercept (%)
A	−2.7 × 10^−3^	52.862	−3 × 10^−3^	58.269	−7.2 × 10^−3^	68.327
B	2.32 × 10^−2^	64.248	2.48 × 10^−2^	66.079	2.33 × 10^−2^	69.857
C	−9.7 × 10^−3^	84.311	1.12 × 10^−2^	86.268	−1.8 × 10^−3^	89.083

**Table 5:** RH (average, intercept, and maximum) measurement for each specimen types in accordance to seasonal conditions.

Specimen Types	Average, Intercept and Maximum of RH Measurement per Seasonal Conditioning								
	Winter (4 °C)			Spring/Autumn (20 °C)			Summer (40 °C)		
	Avg. (%)	Intercept (%)	Max (%)	Avg. (%)	Intercept (%)	Max (%)	Avg. (%)	Intercept (%)	Max (%)
A	51.78	52.86	54.42	57.10	58.27	58.31	65.47	52.86	69.16
B	73.42	64.25	77.79	75.87	66.08	81.64	79.06	69.86	87.85
C	88.14	84.31	90.25	90.69	86.27	92.41	88.38	89.08	93.43

**Table 6:** Comprehensive results.

Temp. Condition (°C)	Evaluation Results														
	Specimen A					Specimen B					Specimen C				
	Avg. (%)	Intercept (%)	Max (%)	Slope	Range (%)	Avg. (%)	Intercept (%)	Max (%)	Slope	Range (%)	Avg. (%)	Intercept (%)	Max (%)	Slope	Range (%)
Winter (4 °C)	51.78	52.86	54.42	−2.7 × 10^−3^	50~70	73.42	64.25	77.79	−2.32 × 10^−2^	52~90	88.14	84.31	90.25	9.7 × 10^−3^	61~95
Spring/Autumn (20 °C)	57.10	58.27	58.31	−3 × 10^−3^		75.87	66.08	81.64	2.48 × 10^−2^		90.69	86.27	92.41	1.12 × 10^−3^	
Summer (40 °C)	65.47	52.86	69.16	−7.2 × 10^−3^		79.06	69.86	87.85	2.33 × 10^−2^		88.38	89.08	93.43	−1.8 × 10^−3^	

## Abbreviations

ASTM	American Standards for Testing and Materials
BS EN	British Standard European
GB	Guobiao
KS	Korean Industrial Standards
LH	KOREA LAND & HOUSING CORPORATION
RH	Relative Humidity
